# Novel Ni/pHEMA-gr-PVP Composites Obtained by Polymerization with Simultaneous Metal Deposition: Structure and Properties

**DOI:** 10.3390/ma12121956

**Published:** 2019-06-18

**Authors:** Oleksandr Grytsenko, Ivan Gajdoš, Emil Spišák, Volodymyr Krasinskyi, Oleh Suberlyak

**Affiliations:** 1 Department of Chemical Technology of Plastics Processing, Lviv Polytechnic National University, 12, St. Bandera str., 79013 Lviv, Ukraine; ogryts@gmail.com (O.G.); vkrasinsky82@gmail.com (V.K.); suberlak@polynet.lviv.ua (O.S.); 2 Institute of Technologies and Materials, Faculty of Mechanical Engineering, Technical University of Košice, 74 Mäsiarska, 04001 Košice, Slovakia; emil.spisak@tuke.sk

**Keywords:** polyvinylpyrrolidone, 2-hydroxyethylmethacrylate, hydrogels, nickel-containing hydrogels, nanoparticles, metal-polymeric composites

## Abstract

The synthesis and study of metal-containing hydrogels, particularly those filled with nickel nanoparticles, is currently of interest to many researchers. This paper presents the results of an investigation of the structure and properties of Ni(0)-filled composites on the basis of 2-hydroxyethylmethacrylate copolymers (HEMA) with polyvinylpyrrolidone (PVP) and their hydrogels. The authors of the article are the first who propose the method to produce these materials by combining the processes of polymer matrix synthesis and a reduction of Ni^2+^ ions. Synthesis is carried out in one stage without complicated equipment and is technologically simple. It is determined by thermometric research that the temperature conditions required for the chemical reduction of Ni^2+^ are achieved due to the heat released during the exothermic reaction of HEMA polymerization in the presence of PVP. With the help of Fourier transform infrared analysis, and thermogravimetric and differential-thermal analysis, the formation of a crosslinked graft copolymer based on HEMA and PVP was confirmed, and its structural parameters, including the efficiency of PVP grafting, PVP content in the copolymer, and the molecular weight of the interstitial fragment of the polymer network, were investigated. The results obtained with scanning electron microscopy revealed that the size of the Ni(0) particles is about 500 nm. X-ray structural analysis of the composites obtained confirmed the existence of metal nickel particles. The strength, elastic, sorption, electrical, and magnetic properties of the obtained composites in the solid (dry) and elastic (swollen) physical states, depending on the composition of the copolymer and the content of the metal filler, have been investigated.

## 1. Introduction

Recently, considerable progress has been made in developing new methods [1] and optimizing the synthesis conditions [2] and properties [3,4] of metal-polymeric composites, which is a prerequisite for the creation of materials with unique properties for various applications [5,6]. To date, there has been widespread interest, both in science and practice, among the metal-polymeric composites in new materials with specific characteristics, known as metal-containing polymeric hydrogels [7]. Polymeric hydrogels are materials with a porous structure [8,9], which, in combination with the presence of hydrophilic functional groups, provides swelling of the polymer matrix in water and other polar solvents [10] that results in their high permeability for dissolved low-molecular substances [11,12]. The uniqueness of the metal-containing hydrogels is that they combine the properties of the hydrogel matrix and the metal filler [13]. The introduction of metal particles into the structure of hydrogels opens additional possibilities for providing them with new properties, for example, bactericidal, antifungal, fluorescent, optical, catalytic, electrical, and magnetic properties, as well as their changes in the desired direction. Such materials are already used in biotechnology and medicine [14]; in the production of packaging materials [15], microelectronics [7], optics and optoelectronics [9], chemical catalysis [16,17], and sensory devices [18]; and in other industries.

Metal-containing hydrogels are distinguished from other metal-filled polymeric materials by their ability to change characteristics, obtained as a result of filling, depending on the temperature, pH, humidity, and content of low-molecular substances. Due to high values of both surface energy and surface area, fine-dispersed metallic particles, especially nanoscale ones, are effective catalysts for many chemical reactions [19,20]. The hydrogel-supported metal particles can be successfully used for the reduction of nitro compounds; the hydrolysis of various hydrides; and the degradation of toxic species, such as dyes, chlorohydrocarbons, pesticides, insecticides, etc. [21]. Nickel-containing hydrogel materials are interesting from both a scientific and practical point of view. Hydrogels, containing particles of Ni(0), are widely used as an effective catalyst, for example, in the reduction reaction of 2- and 4-nitrophenols [22,23] and in the hydrolysis of sodium borohydride in an alkaline medium for obtaining hydrogen [24,25].

The main problem that arises during the production of such materials relates to how to choose the best ways to introduce the filler into the polymer matrix. The size and nature of the filler particles’ distribution in the volume of the composite depend on the filling method, and, consequently, its structure and properties, as well as the productivity and cost-effectiveness of the production. The most common methods employed for the production of such materials are the polymerization of metal powders [26,27] and the precipitation of metal particles in situ directly in the pores of the polymer matrix [28,29,30]. However, filling with powders does not always give the desired result from a technological point of view. During mixing of the composition components, achieving a uniform distribution of metal particles in the hydrogel is not always possible due to their limited compatibility, as well as the possibility of sedimentation, which causes heterogeneity of the composite properties. Therefore, in some cases, it is expedient to directly synthesize metal filler particles in the polymer matrix in turn to prepare homogeneous composites. In such composites, the polymer matrix plays the role of a reactor for the synthesis of metal particles. However, filling by the reduction of metal ions in a network of polymer matrix requires the use of large amounts of oxidizing and reducing agent solutions, which requires an additional stage of regeneration and is accompanied by the over-consumption of reagents. In addition, this method, as well as the other existing methods [31] used for the production of metal-containing polymers, involves at least two steps and contains a step of polymer matrix synthesis and a step of metal-filler particle synthesis.

We have established the opportunity for metal-containing hydrogel synthesis by combining the processes of polymer synthesis and the production of metal particles into one stage.

Copolymers based on 2-hydroxyethyl methacrylate with polyvinylpyrrolidone (pHEMA-gr-PVP) were used to form the polymer matrix. 2-hydroxyethyl methacrylate (HEMA) was selected as the main component of the hydrogel net due to its water solubility, as well as the presence of a carbonyl group that is able to coordinate metal ions [32]. Poly-2-hydroxyethyl methacrylate (pHEMA) and its copolymers are characterized by a high degree of water absorption, the ability to absorb low molecular weight substances, their high biocompatibility, and their low thrombogenicity [33].

Polyvinylpyrrolidone (PVP) has attracted much attention due to its impressive properties, such as its biocompatibility, nontoxicity, solubility in water and many organic solvents, pH stability, affinity to complex both hydrophobic and hydrophilic substances, and status as chemically inert in physiological reactions [34]. The use of PVP opens additional opportunities for the synthesis [35] and stabilization of metal nanopowders [36,37], modification of various substances [38,39] and materials [40,41], improvement of modern technologies [42,43], production of new functional materials [44,45], and the respective expansion of their fields of use [10,46].

A prerequisite for the implementation of the developed method for the production of metal-containing HEMA-gr-PVP copolymers is the high reactivity of HEMA/PVP compositions; possibility of polymerization in the presence of a solvent with the achievement of a high porosity of the polymer matrix, which provides removal of the reduction reaction products from the volume of the composite; and exothermic effects of polymerization, the heat of which can be used to create the necessary temperature conditions for the reduction of nickel ions.

As shown by the results of the study of HEMA and PVP composition polymerization, which occurs with the simultaneous chemical reduction of Ni^2+^, the passing of two chemical processes of different natures has a mutual influence on their rate [47]. In addition to the effect on the rate of polymerization, metal reduction undoubtedly affects the formation of the structure, which, respectively, will determine the properties of the composite. Since the studied composites are new materials, it is necessary to investigate the effect of the developed filling method on their structure and properties for the optimal practical use of these materials.

Given this, the purpose of this work was to investigate the features of the structural formation and properties of nickel-containing composites on the basis of HEMA copolymers with PVP (Ni/pNEMA-gr-PVP), obtained by polymerization with simultaneous Ni^2+^ reduction.

## 2. Materials and Methods

### 2.1. Materials

As a matrix for Ni(0) filling, rare-crosslinked copolymers were selected, which were obtained by the radical polymerization of HEMA in the presence of PVP. Polymerization was carried out with a radical type of initiator– called benzoyl peroxide (BP). HEMA (Sigma Chemical Co) was used after purification and distillation under vacuum (residual pressure 130 N/m^2^, T_boiling_ = 351 K); PVP (AppliChem GmbH) of a high purity with MM 12000 was dried at 338 K in vacuum for 2–3 h before use; and BP inorganic salts (nickel (II) sulfate, sodium hypophosphite, silver nitrate), purified by recrystallization from ethanol, were p.a. grade. The pH of the medium was adjusted with acetic acid and ammonium hydroxide (25%).

The compositions of the structure HEMA:PVP = 90 ÷ 60:10 ÷ 40 mass parts using 10 and 50 mass parts of solvent (Н_2_О) were studied. The lower limit of the PVP content is due to the fact that the time of hardening of the compositions increases significantly (up to 24 h) for its lower content. The upper limit of the PVP content is due to technological complications—the higher content of PVP causes the longer duration of its dissolution in HEMA and the viscosity of the composition increases, which makes it difficult to disperse and deaerate the composition. The need for the presence of the solvent in the original composition was caused by the dissolving reduction precursors in it.

### 2.2. Synthesis Technique of Ni/pHEMA-gr-PVP Composites

The process was carried out at the initial temperature of polymerization of T_0_ = 50 °С in the presence of BP. The reduction of Ni^2+^ during the polymerization was carried out in acidic (pH 4–5) and alkaline (pH 7.5–8) medium. A polymer–monomer composition (PMC) with a BP ([BP] = 0.3 wt.%) initiator was prepared at room temperature, during which the required amounts of BP and PVP were dissolved in HEMA to obtain a homogeneous mixture. Separately, an aqueous solution of oxidant (NiSO_4_) and an aqueous solution of reducing agent (NaH_2_PO_2_) were prepared at the required concentrations. The concentration ratio [NiSO_4_]:[NaH_2_PO_2_] = 1:2.2 (mol/L) was used. In total, 1.3 g/L of AgNO_3_ reducing activator, acetic acid, or 25% ammonia solution was added to the solution of the oxidant to provide the required pH.

PMC, oxidant, and reducing agent solutions were kept separately in a thermostat at the required initial temperature of polymerization before mixing. After the thermostatic treatment, solutions of the oxidizer and the reducing agent were mixed to produce an oxidation-reducing system (ORS), which was immediately mixed with PMC. The mixture obtained was dosed into a polyethylene polymerization mold. The mold provided gas-escape channels for the removal of hydrogen formed during the reduction reaction. After formation, the samples of Ni(0)-containing composites were washed in distilled water to remove unreacted HEMA, PVP, oxidant, and reducing agent, as well as the products of the reduction reaction.

### 2.3. Measurements and Characterization

#### 2.3.1. Standard Methods of Instrumental Research

Attenuated total reflectance Fourier transform infrared (ATR FTIR) spectra were obtained using a PARAGON 1000 FTIR spectrometer (Perkin-Elmer, Norwalk, CT, USA) equipped with a single-horizontal Golden Gate ATR cell (Perkin-Elmer, Waltham, MA, United States). Samples were used in the form of a pellet. The spectra were recorded after 16 scans, at a resolution of 4 cm^−1^, within the range of 500 to 4000 cm^−1^. Differential thermal and thermogravimetric analysis (DTA/TG) was conducted using a derivatograph MOM Q-1500 D (Paulik, Paulik&Erdey, MOM, Budapest, Hungary), using samples of polymers in the form of fine-dispersed powder. The research was carried out in air at a heating rate of 10 °C/min. The temperature range of the investigations was 273–973 К. Measurements were taken using a sample mass of 100 mg. The morphology of samples in a dry state was studied by scanning electron microscope-microanalyzer РЕММА-102-02 (JSC "SELMI", Sumy, Ukraine). The range of the accelerating voltage change was 0.2–40 keV, the range of magnification change was 10–300000, and the resolution was no more than 5.0 nm. The statistical analysis of nickel particles’ distribution by size was realized through measuring the diameter of 200 particles of ten different micrographs for various samples. The measurement error was 15%. The crystal structure of the samples was analyzed by X-ray diffractometry (XRD), for which a DRON-4-07 X-ray Diffractometer (JSC «Bourevestnik», Saint Petersburg, Russia) was used. Irradiating lamps with a copper anode and Ni-filter were used. Investigations were carried out in the range of 2θ from 4 to 360°.

#### 2.3.2. Thermometric Investigations of HEMA Copolymerization with PVP

Thermometric investigations of the polymerization processes of HEMA/PVP compositions with the simultaneous chemical reduction of Ni^2+^ were carried out using a plant, the main element of which was a glass reactor, which, based on its properties, was close to adiabatic. The reactor was previously washed with a chrome mixture and distilled water, dried, and then thermostated at the temperature of the experiment (initial temperature of polymerization). The PMC and ORS were mixed and loaded into the reactor after the thermostat treatment. The reaction volume was 5 ± 0.1 cm^3^. The temperature change of the reaction medium was fixed using a thermocouple with the following representation of the results in the form of the curves of the dependence T = f(τ). The experimental error was 2%.

#### 2.3.3. Efficiency of PVP Grafting

An amount of unbounded PVP was determined using the photocolorimetry of the water extract. The grafting efficiency (*f*, %) was calculated as a ratio between grafted PVP (*m_1_*, g) and the total amount of PVP in the initial blend (*m*, g):

(1) $$f=\frac{m_{1}}{m}\cdot 100,$$

The grafting degree (*p*, %) was calculated as a ratio between grafted PVP (*m_1_*, g) and the total weight of the copolymer (*M*, g):

(2) $$p=\frac{m_{1}}{M}\cdot 100,$$

#### 2.3.4. The Molecular Weight between Cross-Links in the Polymer Network

The molecular weight between cross-links in the polymer network (M_C_, kg/mol) was determined using the Flory–Rehner method [48]. M_C_ was characterized on the basis of the results of studies of the kinetics of swelling of hydrogels of various compositions.

The value of the Flory interaction parameter χ, which describes the interaction between the solvent and the polymer segment, is a convenient indicator for an evaluation of their mutual relationship. In the case of cross-linked polymers, the ratio describing the swelling of the cross-linked polymer in solvent excess is used:

(3) $$\chi V_{2}{}^{2}=-\mathrm{lg}\left(1-V_{2}\right)-V_{2}-\frac{\rho_{2}M_{1}V_{2}{}^{1/3}}{\rho_{1}Mc},$$

where *V_2_* is the volume fraction of the polymer in the swollen network; *ρ_2_* is the density of dry polymer, kg/m^3^; *M_С_* is the average molecular weight between cross-links in the polymer network, kg/mol; *M_1_* is the molecular weight of the solvent; *ρ_1_* is the density of the solvent, kg/m^3^; and *χ* is the Flory solvent–polymer interaction parameter.

Due to the fact that *M_С_* depends on the irregularity of the network, *χ* is determined from the temperature coefficient of the polymer volume fraction:

(4) $$\frac{dV_{2}}{dT}=\frac{\chi V_{2}{}^{2}/Т}{5/3\chi V_{2}+2/3-\left[1/\left(1-V_{2}\right)\right]-\left[\ln\left(1-V_{2}\right)/3V_{2}\right]},$$

The value of the volume fraction of the polymer in the swollen state was determined using the experimentally defined values of the equilibrium degree of swelling *Q_max_*:

(5) $$V_{2}=\frac{1}{1+Q_{\max}},$$

Samples were washed from unreacted residues and dried to a constant weight. Q_max_ was determined by the weight method through the difference of the mass of dry (*m_0_*, g) and swollen to equilibrium state (*m_1_*, g) samples:

(6) $$Q_{\max}=\frac{m_{1}-m_{0}}{m_{0}},$$

The value of *dV_2_/dT* was determined from the obtained straight line dependences *V_2_ = f(Т)*. Substituting *V_2_* and *dV_2_/dT* into Equation (4), we defined *χ*. Using Formula (3) found by *χ*, the *M_С_* was calculated.

#### 2.3.5. Study of the Metal Content Obtained during the Polymerization Process

Samples of a metal-filled copolymer (m = 3 ± 0.3 g) were ground to a micro disperse condition, washed with distilled water, dried to a constant mass, and weighed to an accuracy of 5 × 10^−5^ g. After that, the metal-filled copolymer was etched in dilute nitric acid (50%) during intense stirring. The stirring was carried out at room temperature for 2 h to provide complete interaction of the metal with acid. After etching, the solution was filtered. The filtered etched polymer was washed with distilled water to remove the soluble residues of reaction products of metal with acid, filtered, and dried to a constant weight. The content of the recovered metal in the composite was calculated by changing the mass of the initial copolymer and samples of the copolymer etched in acid.

#### 2.3.6. Physical–Mechanical Characteristics

Deformation and elastic characteristic samples in a swollen state, including the hardness number (*H*, MPa) and elasticity index (*E*, %), were determined using the hardness meter TShR-320 (JSC «Polymermash», Saint Petersburg, Russia) by measuring the difference between the depths of indenter penetration under the action of previous and residual loads [49]:

(7) $$H=\frac{0.1F}{\pi \cdot d\cdot h},$$

(8) $$E=\frac{h-h_{1}}{h}\cdot 100,$$

where *F* is the applied load, N; *d* is the diameter of the indenter ball, mm (*d* = 5 mm); *h* is the depth of ball penetration into the sample under the load *F*, mm; and *h_1_* is the residual deformation after removing the load, mm.

The surface hardness of dry samples (with diameter Ø12 ± 0.1 мм and height *h* = 5 ± 0.01 мм) was found according to the conic yield point in a Höppler consistometer at 293°K by pressing a steel cone with a vertex angle of 58°08′ into a polymeric specimen under a load of 5.0 kg for 60 sec [50]. Boundary water absorption (*W*, wt.%) was determined by the weight method as the difference between dry (*m_2_*, g) and swelled (*m_1_*, g) samples:

(9) $$W=\frac{m_{1}-m_{2}}{m_{1}}\cdot 100,$$

#### 2.3.7. Conductivity

The conductivity characteristics of Ni/pHEMA-gr-PVP composites were estimated by the specific volume resistivity (*ρ_v_*, Ω · m). Samples of a cylindrical shape with a diameter of 12 ± 0.1 mm and a height of 5 ± 0.1 mm were used for *ρ_v_* study. The specific volume resistivity was calculated as

(10) $$\rho_{v}=\frac{R_{v}\cdot S}{h},$$

where *R_v_* is the volume electrical resistance, Ω; *S* is the square of the sample, m^2^; and *h* is the sample thickness, m.

#### 2.3.8. Magnetic Properties

Magnetic characteristics (specific saturation magnetization, coercivity) of Ni/pHEMA-gr-PVP composites were determined using a magnetometer MPMS-XL5 (Quantum Design, San Diego, CA, USA) with a vibrational sample. For analysis, samples of a cylindrical shape with a diameter of 8 mm and height of 3 mm were used, and a standard carbonyl iron was used. 

## 3. Results and Discussion

### 3.1. Synthesis of Ni/рHEMA-gr-PVP Composites

**Scheme 1:**
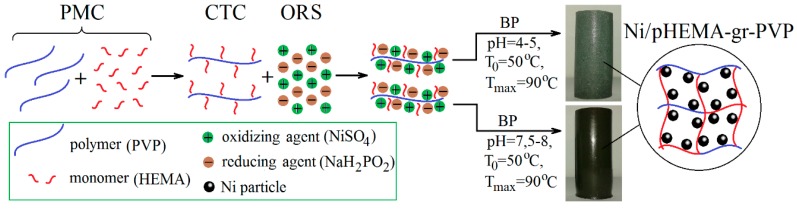
Schematic process of the formation of Ni/рHEMA-gr-PVP composites.

The process of nickel-filled materials based on copolymer pHEMA-gr-PVP production is presented in Scheme 1. The essence of the method is the simultaneous occurrence of HEMA polymerization in the presence of PVP and the reduction of Ni^2+^.

**Scheme 2:**
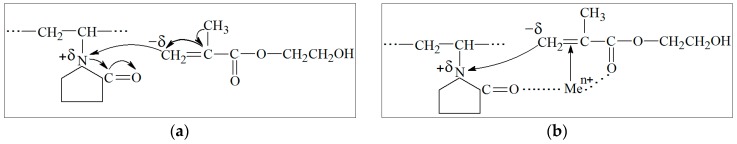
Formation of the charge-transfer complex HЕМА/PVP (**a**) and triple complex HЕМА/Мe^n+^/PVP (**b**).

Mechanisms for the production of pHEMA-gr-PVP copolymers were discussed in our previous works [51,52]. Copolymers were synthesized in a block and solution in the presence of radical polymerization initiators and without them [12,51], under the action of metal ions with a variable degree of oxidation [52] and UV irradiation [10], and in a magnetic field [53]. It was established that the polymerization of HEMA in the presence of PVP occurs through the formation of a charge transfer complex (CTC) with the participation of PVP and HEMA (Scheme 2a). The HEMA molecules are solvated on PVP macromolecules, and the spatial orientation of the first is observed in relation to the other.

**Scheme 3:**
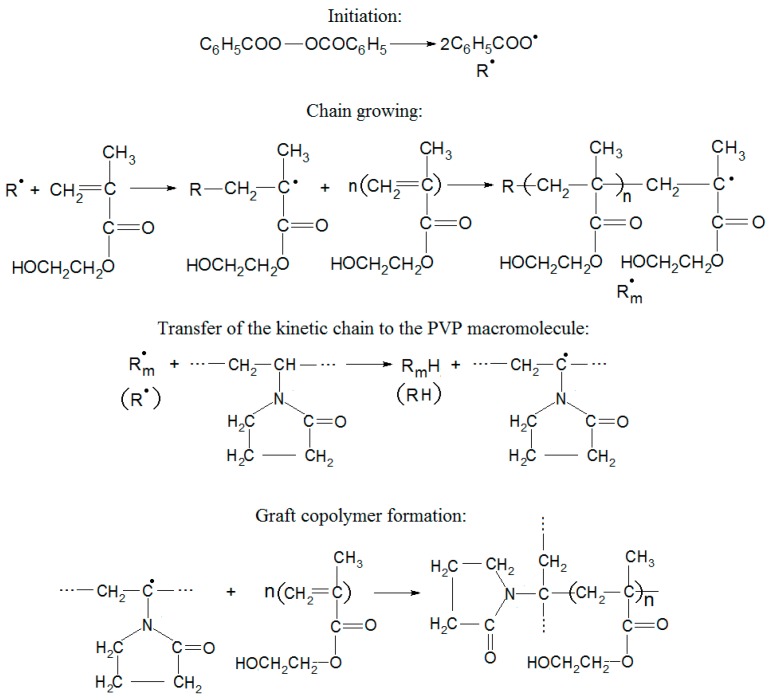
The mechanism of graft copolymer formation.

The formation of a CTC between HEMA and PVP affects not only the kinetics of polymerization, but, more importantly, the structure and composition of the synthesized copolymers [10]. The growth of the HEMA polymerization rate in the presence of PVP is due to the so-called “matrix effect” [10], when the monomer molecules are solvated on the polymer matrix. PVP plays the role of the matrix in this case. Due to the solvation of HEMA molecules on the PVP polymer matrix (Scheme 1 and Scheme 2), it is possible to transfer the kinetic chain to the PVP macromolecule (both from the primary radical R^•^ and from the macroradical R_m_^•^) and the formation of the graft copolymer (Scheme 3) [10].

**Figure 1:**
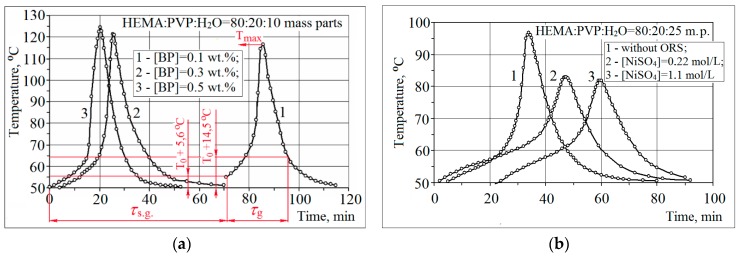
Thermometric curves of polymerization depending on the initiator (benzoyl peroxide (BP)) content (**a**) and oxidation-reducing system (ORS) composition (**b**).

Polymerization of HEMA in the presence of PVP occurs by a radical mechanism and is an exothermic process—it is accompanied by a gel-effect that causes self-heating of the system (Figure 1). Polymerization of HEMA/PVP compositions in the presence of BP is already characterized by a high rate at the initial temperature of 50 °C (Figure 1a). When PMC is added to the metal salts of variable degrees of oxidation, a triple complex HEMA/Me^n+^/PVP (Scheme 2b) [52] is formed. The consequence of the presence of such an interaction is the coordination of Ni^2+^ ions, followed by the stabilization of the formed particles of Ni(0). It has been experimentally proved that the heat released from exothermia causes the reaction of the Ni^2+^ reduction [45]. The kinetic regularities of polymerization were evaluated by the received thermometric curves, which show characteristic parameters corresponding to the start time of the gel formation (τ_s.g._), the time of reaching the maximum exothermic temperature (τ_max.t_), the area of the gel effect (τ_g_), and the maximum exothermic temperature (T_max_).

The presence of even a small ORS content in the initial composition has a significant effect on the rate of the polymer formation process—simultaneous implementation of chemical reduction reactions of metal and polymerization causes the growth of τ_s.g._, τ_max.T_, and τ_g_ and a decrease in T_max_ (Figure 1b). It has been proved that in the presence of a reduction activator and PVP, the reduction reaction already occurs at a high rate at 60 °C [47].

### 3.2. Study of the Ni/pHEMA-gr-PVP Composites’ Structure

**Figure 2:**
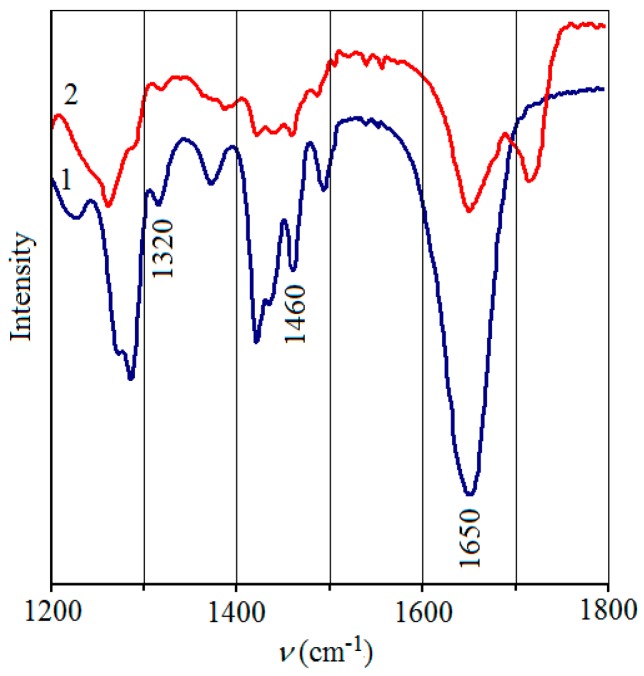
Attenuated total reflectance Fourier transform infrared (ATR FTIR) spectra of polyvinylpyrrolidone (PVP) (**1**) and the Ni/рHEMA-gr-PVP composite (**2**).

In order to confirm the occurrence of the reaction with the transfer of the kinetic chain to the PVP macromolecule and the formation of the graft copolymer during the polymerization of HEMA in the presence of PVP with the simultaneous chemical reduction of Ni^2+^, FTIR spectra of PVP and the Ni/pHEMA-gr-PVP composite extracted with water to the complete removal of unreacted PVP (Figure 2) were obtained and analysed.

The analysis of FTIR spectra shows that in the spectrum of the Ni-filled copolymer, there are typical PVP bands in the ranges 1320 cm^−1^, 1460 cm^−1^, and 1650 cm^−1^ [54]. This fact indicates the presence of PVP units in the copolymer. The depletion of hydrogen from tertiary carbon during the graft polymerization is indicated through the change in the absorption band intensity in the range 1318–1320 cm^−1^ (Figure 2), which significantly decreases in the case of the transition from PVP to extracted filled copolymer and is typical for deformation oscillations of the С–H group of the carbon chain. In addition, on the IR spectrum of the copolymer, a significant decrease in the intensity of the characteristic PVP band in the 1372 cm^−1^ range is observed, which points to the wagging vibration of the CH_2_–CH link of polyvinylpyrrolidone, proving the PVP tertiary carbon participation in the grafting reaction (Scheme 3).

**Figure 3:**
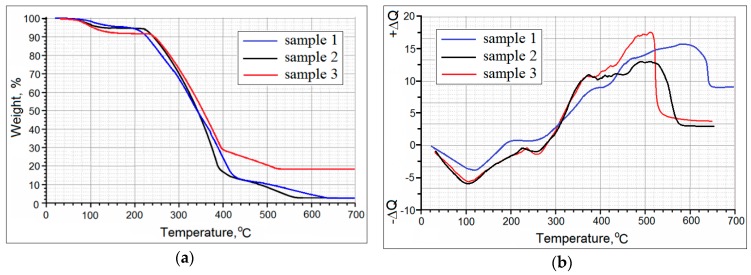
Thermogravimetric (**a**) and differential-thermal (**b**) analysis of (co)polymers: **sample 1** – Ni(0)/рHEMA/PVP mixture (рHEMA:PVP = 80:20 m.p., [Ni] = 1,5 wt.%); **sample 2** – Ni(0)/рHEMA-gr-PVP composite (HEMA:PVP = 80:20 m.p., [Ni] = 1,5 wt.%); **sample 3** – Ni(0)/рHEMA-gr-PVP composite (HEMA:PVP = 8:20 m.p., [Ni] = 16 wt.%).

HEMA grafting to PVP with the formation of a crosslinked copolymer and the effect of the reduced metal content on the thermal stability of composite polymers were confirmed through comparing the results of thermogravimetric (TG) and differential-thermal (DTA) analysis (Figure 3).

A mixture of homogeneous pHEMA and PVP homopolymers with previously obtained Ni(0) powder (sample 1) and Ni/pHEMA-gr-PVP composites with different filler contents (samples 2,3) have been investigated. As can be seen from the obtained TG analysis curves, in the first stage, there is a slight loss of sample mass connected with the evaporation of physically-bounded moisture (up to 200 °C). The intense weight loss of the homopolymer mixture (Sample 1) starts at approximately 200 °C, while samples 2 and 3 begin to lose weight at 220 °C and 235 °C, accordingly.

The loss of the main weight of samples occurs in the temperature range of 200–440 °C. In this temperature range, sample 1 (Figure 3a) loses 81 wt.% of weight, and samples 2 and 3 lose 81 wt.% and 67 wt.%, respectively. The smallest weight loss during the destruction of sample 3 and the highest temperature of destructive processes indicate the effect of the higher content of the reduced metal on the formation of a dense physical network with metal in the nodes. At the same time, on the DTA curve of sample 1, in the range of temperatures of 180–220 °C, a peak of the exoeffect is observed, followed by the insignificant absorption of heat (Figure 3b). The exothermic effect, which can be observed in the temperature range of 180–220 °С, corresponds to the process of the thermooxidation of weakly bound groups of the polymer chain, followed by the degradation and release of volatiles.

However, on the curves of the DTA copolymers, the exothermic peak in the range 180–220 °C is shifted by 40 °C into the region of 220–260 °C. The exoeffect of oxidation on the DTA curve, as well as the beginning of intense weight loss at higher temperatures, indicates the higher thermal stability of the metal-filled copolymers compared to the homopolymers mixture. The results can be explained by the lower content of tertiary hydrogen atoms in the fragments of the PVP chain in the copolymer (Scheme 3), compared to the content of tertiary hydrogen atoms in PVP macromolecules, as well as the participation of the formed metal in the formation of a stable physical net.

The endoeffect in the temperature range 220–280 °C of samples 2 and 3, in comparison with sample 1, is deeper and shifted to the range of higher temperatures. This indicates the formation of more crosslinked copolymer structure involving oxidized ≡C–H groups. For the mixture of pHEMA and PVP (sample 1), the smallest value of the endoeffect is typical, which is shifted to the range of lower temperatures (Figure 3b) and the maximum exoeffect of combustion.

Comparing the rate of weight loss of samples with different reduced metal contents, we can see that the copolymer with the bigger content of nickel (sample 3) is characterized by a higher thermal stability. Therefore, we can affirm that the particles of the obtained Ni obviously affect the copolymer structuring, due to the localization of the monomer molecules in the field of the polarized Ni^2+^/PVP complex (Scheme 2b), as well as the involvement of Ni in the formation of a physically-stable network.

On the DTA curves of copolymers, there are exothermic peaks in the temperature range 360–400 °C. Since, in this temperature range, there is no increase in the weight of samples, these exoeffects may be related to the combustion of the organic phase, as well as the process of nickel crystallization [54]. The change in the slope of the DTA curve of sample 3 after the mentioned exoeffect is due to the phase transition and is connected with the loss of ferromagnetic properties of nickel (Curie point). For nickel, the Curie point is T_C_ = 358 °C [55]. The increase in temperature above 400 °C is accompanied by the appearance of an exoeffect on the DTA curve in the temperature range of 460–520 °C, which indicates the oxidation of nickel along with the combustion of organic matter.

**Figure 4:**
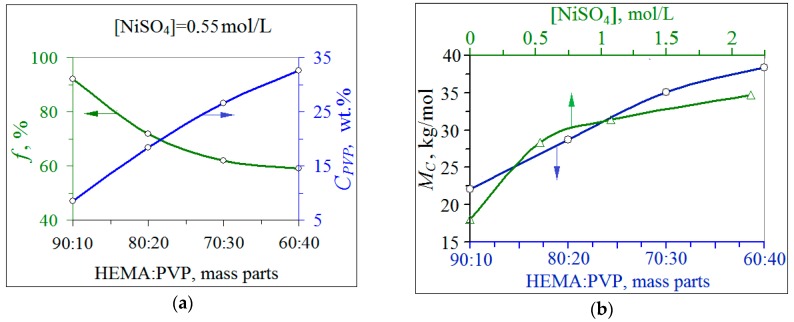
Structural characteristics of Ni/рHEMA-gr-PVP composites: (**а**) – effect of initial polymer–monomer composition (PMC) composition on grafting efficiency (*f*) and polyvinylpyrrolidone (PVP) content in co-polymer (*C_PVP_*); (**b**) – effect of initial PMC composition ([NiSO_4_] = 0.55 mol/L) and concentration [NiSO_4_] in the oxidation-reducing system (ORS) (HЕМА:PVP:S_ORS_ = 80:20:50 m.p.) on the molecular weight between cross-links in the polymer network (M_C_).

Additional confirmation of the graft copolymer formation is seen in the results of research on the graft PVP amount in the copolymer. Previous studies [10] showed that the presence of PVP in the copolymers of alkylmethacrylates gives them increased hydrophily, heat resistance, and mechanical properties, compared to homopolymers. However, not all PVP enters the grafting reaction. As a result, there is discrepancy between the content of PVP in the original composition and in the copolymer. Therefore, the structure of the copolymers was characterized by parameters such as the efficiency of the grafting of the PVP (f); the composition of the copolymer, that is, the ratio pHEMA:PVP in the copolymer; and the density of the crosslinking of polymer network, characterized by the molecular weight between cross-links (M_C_, kg/mol). The structural characteristics of the obtained copolymers and the composition of the copolymer significantly depend on the extent of the ratio of HEMA:PVP in the initial composition—with the increase of PVP content in the PMC, its amount increases in the copolymer (Figure 4a). However, in this case, f decreases significantly, which results in the formation of more porous material with a lower degree of crosslinking (Figure 4b), which results in the washing of unreacted PVP.

The decrease in the density of the copolymer structural network is connected with the influence of the PVP (Figure 4b), in which macromolecules play a role as a peculiar loosener in the network—there is the formation of macro-radicals of a considerable size during the transfer of the chain to the polymer (Scheme 3), which increases the chain length between cross-link points. In addition, part of the PVP that does not participate in the grafting reaction during hydration is washed out to form microcavities in the volume of the polymer. Analyzing the obtained results, it should be noted that the degree of crosslinking of the obtained copolymers is much less than that of the non-filled copolymers [52] and composites obtained by the polymerization filling with nickel powder [49]. This phenomenon is explained by the formation of additional cavities in the composite after washing of the unreacted components of the ORS and the products of the reaction of reduction. With an increase of the oxidizer content in the composition, Mc increases (Figure 4b).

**Figure 5:**
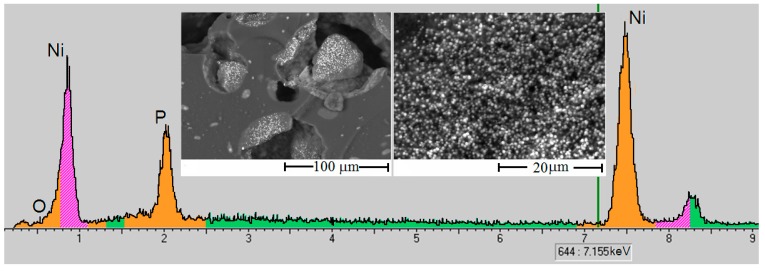
Scanning electron microscopy (SEM) images and energy dispersion analysis (EDA) histograms of the Ni/рHEMA-gr-PVP composite (HЕМА:PVP:S_ORS_ = 80:20:25 mass parts; [NiSO_4_] = 1.1 mol/L; pH = 4.5).

It has been established that the combination of two processes, including polymerization and reduction, in addition to influencing the formation of the structure of the polymer matrix, affects the nature of the distribution and structure of nickel particles. When polymerization with reduction is performed in acidic medium, typical homogeneity of the distribution of nickel particles in pores and their monodispersity can be noted (Figure 5).

Obviously, this kind of filling is ensured by the formation of the proposed triple complex HEMA/Me^n+^/PVP (Scheme 2b) with the subsequent stabilization of Ni(0) by the polymeric matrix. In terms of the form, all particles are spherical, with an average diameter of 500 nm.

**Figure 6:**
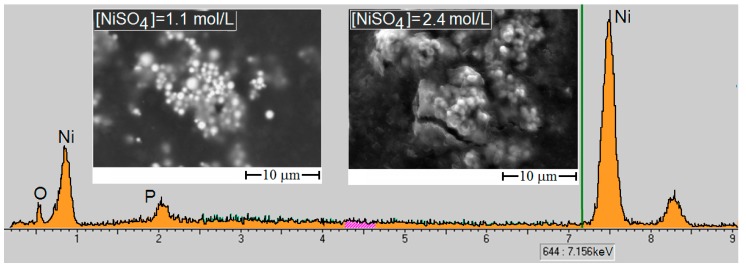
Scanning electron microscopy (SEM) images and energy dispersion analysis (EDA) histograms of the Ni/рHEMA-gr-PVP composite (HЕМА:PVP:S_ORS_ = 80:20:25 mass parts; рН = 8).

During the process in the alkaline medium, the distribution of metal particles in the volume of the material is nonuniform (Figure 6), and the size of the metal filler particles is within the range of 0.5–1 μm and depends on the concentration of the oxidizer.

**Figure 7:**
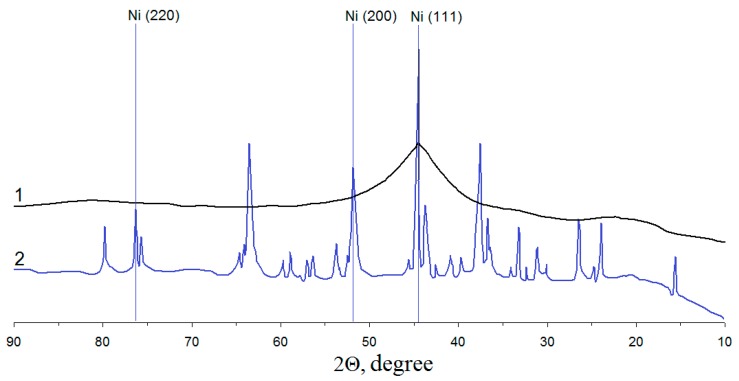
X-ray diffractometry (XRD) patterns of Ni/рHEMA-gr-PVP composites before thermal treatment (**1**) and after thermal treatment (**2**).

As the concentration of the oxidant increases, the amount of reduced Ni^2+^ increases (Figure 6). It should be noted that the composition of the surface of nickel particles depends on the pH of the medium. As the results of the energy dispersion analysis (EDA) show, the surface of nickel obtained in the acidic medium is characterized by a higher content of phosphorus (Figure 5) and, at the same time, a lower content of Ni oxide than the surface of nickel reduced at pH 8 (Figure 6). The results obtained with SEM do not allow us to affirm that the production of Ni(0) occurs due to chemical regeneration during polymerization. X-ray diffraction analysis of the composites was carried out for additional confirmation of the metal filler formation (Figure 7).

However, after analyzing the obtained XRD pattern, it can be noted that there are no peaks on the curve that correspond to Ni(0) (Figure 7, curve 1). However, another confirmation of the reduction of metallic nickel is the magnetic susceptibility of the composites obtained under the II regime. It is possible to assume that during the reduction with simultaneous polymerization, an X-ray-amorphous metal is formed that does not appear on the XRD pattern. X-ray structural analysis of samples after their heat treatment was carried out to confirm this assumption. Heat treatment was carried out for 2 h at a temperature of 500 °C. After recrystallization, the peaks that appeared are typical for Ni(0) (Figure 7, curve 2). Taking into account the fact that the samples for analysis were washed from the residues of the oxidizing agent, the reducing agent, and the reaction products of the reduction, it can be concluded that the peaks, typical for Ni(0), appeared after recrystallization of the X-ray amorphous metal.

### 3.3. Properties of Metal-Filled Ni/pHEMA-gr-PVP Copolymers

Polymer hydrogels are distinguished from other materials because they have limited swelling abilities in water and aqueous solutions of various substances. However, without access to moisture, these materials are in a vitreous state. Therefore, the properties of Ni/pHEMA-gr-PVP copolymers were studied, respectively, in the elastic (swollen) and solid (dry) states. The simultaneous implementation of two processes, including polymerization and Ni^2+^ reduction, has a mutual influence on the properties of the metal filler and polymer matrix and composite in general. It has been established that the properties of Ni/pHEMA-gr-PVP composites greatly depend on the conditions of Ni^2+^ reduction.

**Figure 8:**
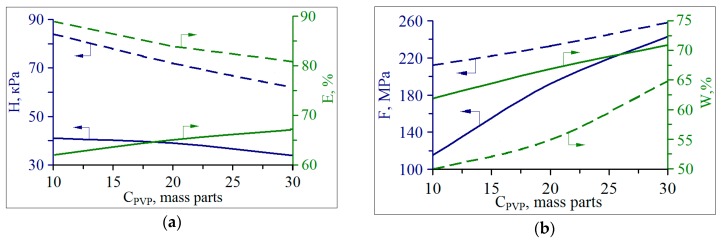
Effect of polyvinylpyrrolidone (PVP) content in a polymer–monomer composition (PMC) on the properties of Ni/рHEMA-gr-PVP composites, obtained at pH 4 (–––) and at pH 8 (-----) in swollen (**а**) and dry (**b**) states ([NiSO_4_] = 0.55 mol/L).

Samples of composites synthesized at initial pH 7.5–8 have higher strength properties, both in dry and swollen states, but are characterized as having a lower water content than samples obtained at the initial pH 4–4.5 (Figure 8).

An increase in the content of PVP in the initial PMC results in a decrease of the strength and elastic characteristics of swollen copolymers (Figure 8a). This is evidently due to the fact that during the hydration part, PVP macromolecules are washed away from the copolymer and do not accept the applied load, and due to the increase in free volume, the possibility of conformational chain changes due to external mechanical forces increases. Due to this, more PVP macromolecules are contained in the unit volume of the copolymer, the polymer is looser, and its hardness and elasticity in the hydrated state are lower. At the same time, the increase in the content of PVP in the initial composition increases the surface hardness F of dry specimens and their ability to swell, so the water content W increases (Figure 8b).

**Figure 9:**
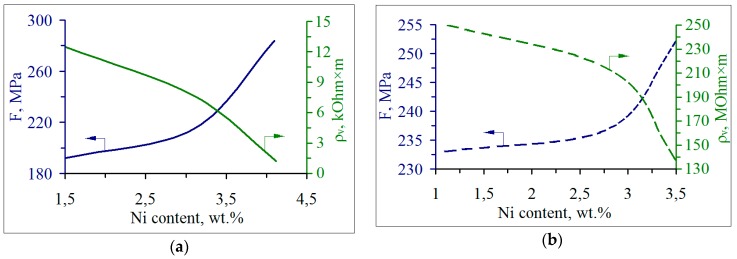
Effect of Ni(0) content on surface hardness (F) and specific volume electrical resistance (ρ_V_) of dry composites, obtained at pH 4 (**a**) and at pH 8 (**b**).

As the content of metal filler increases, the strength characteristics of the composites increase (Figure 9). The content of Ni(0) was regulated by changing the concentrations of oxidizer and reducing agent.

Non-filled pHEMA-gr-PVP copolymers in a dry state are dielectrics. One of the consequences of their filling with Ni(0) particles is the ability to conduct an electric current. An improvement of electrical conductivity is observed with an increase in the content of reduced Ni^2+^ in the composite (Figure 9). It can be noted that composite copolymers obtained in an acid medium are characterized by an electrical conductivity that is 10^4^ times higher than that obtained in alkaline medium (Figure 9).

At the same time, it was found that the composites obtained in the alkaline medium obtain magnetic properties. The combination of the magnetic properties of the filler and the sorption ability of the hydrogel matrix is a prerequisite for the creation of up to date, new materials, named magnetic sorbents, that are capable of structural changes under a magnetic field [56].

**Figure 10:**
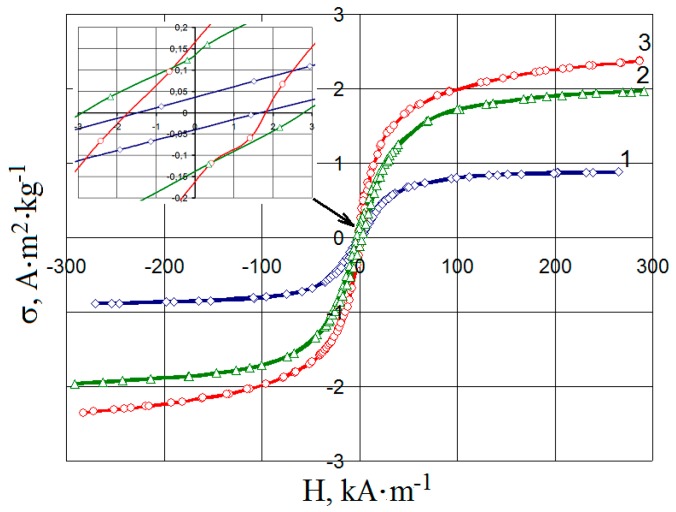
Magnetic hysteresis loops of Ni/рHEMA-gr-PVP composites with different concentrations of NiSO_4_ in the oxidation-reducing system (ORS) (HЕМА:PVP:S_ORS_ = 80:20:25 mass parts): **1** – [NiSO_4_] = 0.55 mol/L; **2** – [NiSO_4_] = 1.1 mol/L; **3** – [NiSO_4_] = 2.32 mol/L.

In order to characterize the magnetic properties of the studied materials, their magnetization curves were obtained and studied (Figure 10), from which the value of magnetization (σ, A·m^2^·kg^−1^) and the coercive force (H, kA·m^−1^) were determined.

From the analysis of the curves’ shape and the small amount of coercive force (within 1.4 ÷ 2.8 kA·m^−1^), it follows that the investigated materials are magnetized. This is important in view of the fact that in a variable magnetic field, the transformation of magnetic energy into heat is rather small. Products based on Ni/pHEMA-gr-PVP composites in a variable magnetic field will be characterized by minimum energy losses per cycle.

Magnetic properties of the Ni/pHEMA-gr-PVP composites have been investigated after their hydration in water and drying. Therefore, preservation of the magnetic properties of the samples proves that the Ni(0) particles have not oxidized and did not change their structure and properties over time, which is additional confirmation of the stabilizing action of the polymer matrix.

## 4. Conclusions

Composite metal-filled materials based on HEMA and PVP copolymers were produced by the method of combining the processes of polymer matrix synthesis and Ni^2+^ ion reduction. The basis of the proposed method is the use of the exothermia of the polymerization process to provide the necessary temperature conditions for the reaction of Ni^2+^ chemical reduction. This approach is particularly attractive from both a practical and scientific point of view, since Ni(0) particles are formed simultaneously during the formation of the polymer matrix, which makes it possible to achieve a better, uniform distribution of particles and to obtain material with isotropic properties. The course of graft polymerization of HEMA on PVP with the formation of a crosslinked copolymer is confirmed.

The composite materials obtained are characterized by a homogeneous structure, filled with nano- and ultrafine Ni(0) particles, the size of which, depending on the technological regimes of synthesis, is within the range of 0.5–1 μm.

The interconnection of physical and mechanical properties of synthesized composites with their structure is determined. The produced materials have high-strength and elastic properties, which depend on the content of PVP in the initial composition and the concentration of the obtained filler. The water content during equilibrium swelling, depending on the components of the composition and the regime of synthesis, is 50 ÷ 71 wt.%. Talking about electrical conductivity, the obtained materials relate to conductors. The specific volumetric resistance of materials obtained in the acidic medium is within the range of 10^3^ Ohm · m, which is 10^4^ times higher than the resistance of samples obtained in alkaline medium, which, in turn, have magnetic properties.

In the future, further research is planned in the direction of creating conditions for reducing the size of metal-filler particles, as well as testing obtained materials as catalysts of chemical processes.
